# Models and key elements of integrated perinatal mental health care: A scoping review

**DOI:** 10.1371/journal.pmen.0000164

**Published:** 2025-03-17

**Authors:** Michelle Carter, Angela Russolillo, Christine Ou, Enav Z. Zusman, Wendy A. Hall, Iva W. Cheung, Emily Jenkins

**Affiliations:** 1 School of Nursing, University of British Columbia, Vancouver, Canada; 2 St. Paul’s Hospital, Providence Health Care, Vancouver, Canada; 3 Department of Obstetrics & Gynaecology, University of British Columbia, Vancouver, Canada; 4 School of Nursing, University of Victoria, Victoria, Canada; PLOS: Public Library of Science, UNITED KINGDOM OF GREAT BRITAIN AND NORTHERN IRELAND

## Abstract

Perinatal mental illness is a common and significant complication of pregnancy and childbirth. When left untreated, these illnesses are associated with an increased risk of adverse health outcomes for mothers, infants, and families. While early detection and effective management are essential, less than 15% of affected individuals receive timely and appropriate treatment. Integrated care offers a promising approach to addressing complex treatment barriers; however, the core features of integrated perinatal mental health (PMH) care are not well understood. We conducted a scoping review to identify and synthesize evidence on existing models and key elements of integrated PMH, with data extracted according to PRISMA guidelines. The search was conducted across four databases: Ovid MEDLINE, EMBASE, PsycInfo, and CINAHL. We included peer-reviewed articles, published in English between 1990 and 2024, that described models of integrated PMH care. Three reviewers independently screened 4588 articles by title and abstract, with 153 articles selected for full-text review. A total of 45 peer-reviewed articles were retained for analysis. These articles described a wide range of integrated PMH care models, including specialized inpatient units, intensive hospital day programs, outpatient and community clinics, and collaborative and stepped-care frameworks. An analysis of these models identified seven key elements of integrated care: (1) screening, assessment, and triage; (2) integrated care delivery; (3) patient-centred care; (4) a biopsychosocial approach to treatment; (5) PMH-trained clinicians; (6) health promotion and illness prevention; and (7) transition and discharge planning. This evidence suggests that care integration improves the accessibility, continuity, and quality of PMH care. Integrated models of care can take many forms with positive impacts on perinatal individuals and their families. Research is needed to establish consensus on the key elements of integrated care to support implementation.

## Introduction

Integrated care refers to the diverse set of frameworks and initiatives that facilitate improvement in health outcomes, patient experiences, and use of resources through intentional coordination and continuity of care [1–3]. In the context of increasingly siloed healthcare services, integration offers a promising approach to improving treatment access, care quality, and clinical communication, particularly in the management of individuals with complex health and social needs [1,4,5]. Despite growing evidence to support the effectiveness of integrated care [1,4,5], Canadian perinatal mental health (PMH) services remain largely fragmented and underdeveloped. This health service gap presents a critical challenge in the delivery of comprehensive and coordinated care for perinatal people and their families [6–8].

Perinatal mental illness (PMI) is a significant complication of childbirth, affecting up to 1 in 5 pregnant and postpartum individuals [9,10]. These illnesses include a variety of conditions, most commonly depression and anxiety, but also bipolar, psychotic, and obsessive-compulsive disorders [9,10]. When left untreated or undertreated, PMIs increase the risk of obstetrical complications, future mental health challenges, and decreased quality of life [11,12]. PMIs are also associated with insecure infant attachment, impaired infant development, and in rare cases, maternal suicide and infanticide [12–14]. Despite the potentially serious consequences of illness, treatment rates remain low. Fewer than 15% of individuals with PMIs receive treatment and, when available, timely treatment is often not achieved [15–20].

Challenges in diagnosing and managing PMIs add another layer of complexity to PMH care. Many health care providers have difficulty differentiating expected distress associated with pregnancy and postpartum adjustment from PMI [21,22]; overlapping obstetrical recovery and mental health concerns can make somatic symptoms (e.g., fatigue, sleep disturbances, low energy) difficult to interpret and treat [23]. To manage the challenges, it has been proposed that PMIs would benefit from integrated care to disentangle their features and transcend a disease-oriented approach [23]. Integration would ideally address clinical recommendations from multiple health care providers while supporting patients’ preferences, values, and capabilities [4,9]. Despite evidence suggesting that improved social and health outcomes would be likely if PMH services adopted a more coordinated and patient-centred approach to care [24,25], ideal processes and outcomes have been difficult to achieve given current limitations in the PMH care system.

In Canada, PMH services face significant challenges rooted in long-standing systemic issues, including unequal access to services, long waitlists, and inconsistent screening policies [26]. In the absence of a national PMH strategy and evidence-based PMH policies, many individuals with PMIs are never screened or assessed [8,27]. For individuals who are identified, care is often constrained by the limited availability of specialized PMH services and adequately trained clinicians [6–8]. Provider silos in the health care system further reduce access to PMH services. When mental health, obstetrical, and primary care teams work in isolation [7,21], communication, coordination, and care processes can become fragmented, with negative impacts on patient safety, care continuity, and ultimately, health outcomes [28–31].

Given the need to improve access, coordination, and quality of PMH services in Canada, we conducted this scoping review with the aim of identifying models and key elements of integrated PMH care. Here, we define *models* as existing frameworks or real-world programs and *elements* as characteristics of integrated care strategies.

## Methods

Scoping review methodology provides a useful approach to describe the nature and breadth of research in a relevant field; summarize a body of findings; and identify research gaps [32]. We used Arksey and O’Malley’s [32] five-stage framework and were further informed by Levac and colleagues’ [33] insights, with data extracted according to PRISMA guidelines [34]. Our search was initially conducted in 2021 and updated in 2024 in preparation for publication. Our approach is detailed below.

### Stage 1. Identifying the research question

The purpose of this review was to review and synthesize the current knowledge regarding integrated PMH care. The research question that informed this scoping review was: *What models and key elements of integrated PMH care are articulated in the existing literature?*

### Stage 2. Identifying relevant studies

Using a search strategy developed in consultation with a reference librarian, we searched four academic databases: Ovid MEDLINE, EMBASE, PsycInfo, and CINAHL. Search terms included relevant Medical Subject Heading (MeSH) terms and keyword synonyms for ‘perinatal,’ ‘mental health,’ ‘integrated care,’ and utilized a search strategy developed and piloted in EMBASE and subsequently modified for use in the remaining databases. The search strategy (see Supplemental 1) was designed to facilitate a wide and thorough investigation of the literature, which included clear criteria to guide article selection (see Table 1).

**Table 1:** Inclusion and exclusion criteria.

Inclusion criteria	Exclusion criteria
Studies describing a model of integrated perinatal mental health care	Studies that do not relate to the perinatal period (i.e., pregnancy and up to the first 12 months following birth), *mental* health, *maternal* mental health, and integrated models of care Studies that address mental health concerns involving individuals other than the birthing parent (e.g., partner or family mental health)
Peer-reviewed empirical studies, program descriptions, and systematic reviews	Opinion pieces, position papers, commentaries, conference abstracts, and editorials
Studies published between 1995 and 2024	Studies published before 1995
English language studies	Studies not available in the English language

### Stage 3. Study selection

**Fig 1:**
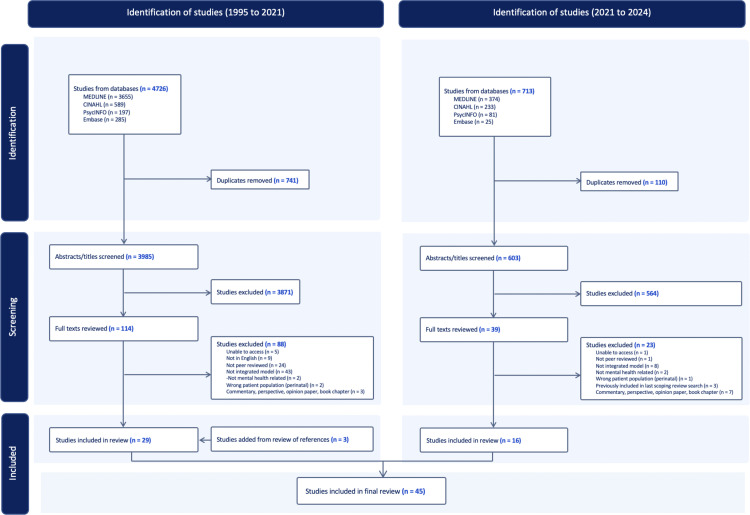
PRISMA flowchart of included articles.

Articles were retrieved across the four databases and uploaded to Covidence, an online reference management software, to facilitate review [35]. The initial search produced 4726 articles (published between 1995 and 2021), of which 336 were duplicates, leaving 3985 for screening. Two reviewers (MC, AR) independently screened titles and abstracts, excluding a total of 3858 articles based on pre-defined selection criteria (Table 1). Discrepancies regarding inclusion were resolved through consultation with a third reviewer (EJ). The remaining 114 full-text articles were read independently, and a further 88 articles were excluded. This resulted in 26 relevant studies being included in the review. Reference lists of the included articles were reviewed by a research assistant, resulting in the addition of three articles. The updated search produced 713 articles (published between 2021 and 2024), of which 110 were duplicates, leaving 603 for screening. After title, abstract, and full-text reviews conducted independently by two reviewers (MC, IC), an additional 16 articles were added for analysis (Fig 1).

### Stage 4. Charting the data

We retained a total of 45 articles for analysis. The team developed a structured data extraction table to capture study characteristics and information to support the interpretation and contextualization of findings [32,33]. This template was developed iteratively, with categories added to best address the research question [33]. Categories comprised general study details, such as aim, geographic location, design, population of interest, care setting, and team composition. Additionally, intervention details and descriptions of care model components were documented. Four reviewers (MC, AR, CO, IC) extracted data, and a research assistant screened data for accuracy.

### Stage 5. Collating, summarizing, and reporting the results

Descriptive statistics (e.g., frequency counts, percentages, etc.) were calculated to summarize the characteristics of included studies. Critical content analysis was used to synthesize extracted information and generate thematic categories capturing key insights about care models and core elements of integrated PMH care [36].

## Results

### Study characteristics

**Fig 2:**
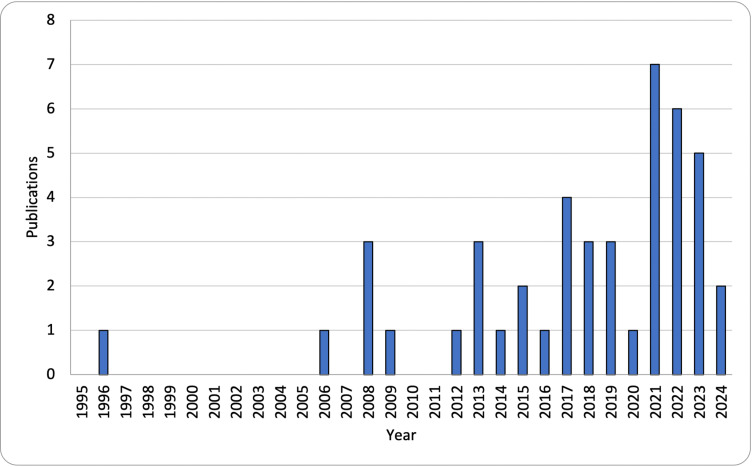
Articles by year (n = 45).

This scoping review included 45 articles published from 1996 to 2024. Fig 2 illustrates the recent growth in this field, with 39 (87%) articles published in 2012 or later. The characteristics of the included articles are summarized in Table 2.

**Table 2:** Characteristics of included articles (n = 47).

	n (%)
**Geographical focus**	
United States	22 (48.9)
Australia	5 (11.1)
United Kingdom	3 (6.7)
Canada	2 (4.4)
Nigeria	2 (4.4)
Germany	1 (2.2)
Uganda	1 (2.2)
Ireland	1 (2.2)
South Africa	1 (2.2)
Japan	1 (2.2)
Mali	1 (2.2)
Multi-national	5 (11.1)
**Study type**	
Program description	18 (40.0)
Quantitative	13 (28.9)
Evidence syntheses	5 (11.1)
Survey analyses	3 (6.7)
Qualitative	3 (6.7)
Mixed methods	3 (6.7)
**Mental health concern**	
Depression only	19 (42.2)
Depression and anxiety	11 (24.4)
Severe PMIs^*^	8 (17.8)
Other	7 (15.6)
**Perinatal stage**	
Preconception, pregnancy, and postpartum	2 (4.4)
Pregnancy and postpartum	32 (71.1)
Pregnancy only	5 (11.1)
Postpartum only	6 (13.3)

*Severe PMIs broadly describe mental illnesses that substantially impact maternal functioning and include a range of conditions, including bipolar and psychotic disorders.

Eleven countries were represented in the included literature, with most articles originating from the United States (22, 48.9%), Australia (5, 11.1%), or the United Kingdom (3, 6.7%). The remaining articles were led by teams from Canada (2, 4.4%), Nigeria (2, 4.4%), Germany (1, 2.2%), Uganda (1, 2.2%), Ireland (1, 2.2%), South Africa (1, 2.2%), Japan (1, 2.2%), and Mali (1, 2.2%), with five articles (11.1%) reporting data from multiple countries (Table 2).

A large proportion of the literature either described existing PMH care models (18, 40.0%) or evaluated PMH care models through quantitative methods (13, 28.9%), including randomized control trials (5, 11.1%), longitudinal cohort designs (3, 6.7%), and pre-post intervention evaluation such as through chart reviews (5, 11.1%). Other articles used evidence syntheses (5, 11.1%), observational survey analyses (3, 6.7%), qualitative methods (3, 6.7%), or multiple or mixed methods (3, 6.7%) to examine PMH programs or to offer practical suggestions to inform service design and delivery (Table 2).

Perinatal depression emerged as the focus of integrated care across publications (11, 42.2%). Most articles (32, 71.1%) focused on both pregnancy and postpartum periods, with two (4.4%) also addressing preconception. Definitions of the postpartum period varied across studies, ranging from one month to two years following birth (Table 2).

### Models of integrated PMH care

Table 3 provides an overview of the models and key elements of integrated PMH care that were captured in this scoping review.

**Table 3:** Overview of included articles (n = 47).

Author (Year)	Country	Aim	Study Design	Perinatal Stage	Mental Health Concern	Care Model	Key Care Elements
Baker-Ericzén et al. (2008) [37]	United States	“To describe a collaborative program for addressing maternal depression in the postpartum period.”	Prospective cohort study	Postpartum	Depression	Collaborative care (*Partnership for Women’s Health*) with proactive follow-up from a mental health advisor	Screening, assessment, patient education, mental health advisors, counselling, mental health referral process
Beaumont et al. (2022) [38]	Australia	“To test a model of care providing step up/step down support to women with moderate-severe perinatal mental health disorders awaiting hospital admission.”	Chart/administrative data review for patients; survey to capture clinician experience	Postpartum	Moderate to severe mental health problems (e.g., depression, bipolar affective disorder, psychosis, schizophrenia))disorders	Stepped care, home-based supports	Screening, assessment, community-based supports, admission if needed, post-discharge support
Bowen et al. (2008) [39]	Canada	“To describe the Maternal Mental Health Program, a shared care program that co-locates multidisciplinary clinicians within a primary care setting.”	Program description	Preconception, pregnancy, and postpartum	Depression and anxiety	Collaborative care (*Maternal Mental Health Program*) with co-location of mental health and primary care providers	Co-location of mental health and primary care providers, Individual/group therapy, support groups, psychoeducation, assessment, treatment, crisis intervention, community referral process, on-site childcare
Cooney et al. (2023) [40]	Ireland	To evaluate the impact of the introduction of Specialist Perinatal Mental Health Services on prescribing practices and treatment pathways in an Irish maternity hospital.	Pre- and post- program chart review	Pregnancy and postpartum	Depression	Collaborative care (multidisciplinary outpatient care and liaison ward-based inpatient care)	Assessment; patient education; clinical nurse specialist individual support; cognitive behavioral therapy; midwife support for mental health, bereavement, and birth reflection; psychotropic medication
Cox et al. (2017) [24]	United States	“To describe the development of an integrative model of care in an obstetrics and gynecology setting that was expanded to a specialized perinatal psychiatric inpatient unit.”	Program description	Pregnancy and postpartum	Depression and anxiety	Inpatient Unit and collaborative care with psychiatric NP embedded in clinic	Patient engagement, screening, assessment, treatment algorithm, medications, psychoeducation, psychological interventions, family support, lactation support, recreational therapy, PMH-trained staff, co-location of mental health NP and maternity providers
Geller et al. (2018) [41]	United States	“To present an overview of Mother Baby Connections (MBC) and clinical outcome data.”	Survey	Pregnancy and postpartum	Mood and anxiety disorders	Intensive day program (*Mother Baby Connections*)	Screening, assessment, care coordination meetings, staff training, referral process, patient navigator, onsite childcare, psychological interventions (e.g., CBT, IPT, DBT, ACT), mother-baby therapy, recreational groups, psychoeducation, pharmacotherapy, couples’ therapy, EMDR
Gjerdingen et al. (2009) [42]	United States	“To pilot a stepped collaborative intervention for women with postpartum depression, and evaluate health differences between women with self-diagnosed depression and no depression.”	RCT	Postpartum	Depression	Stepped care	Screening, assessment, primary care treatment, mental health consultation, referral process, stepped care treatment options, psychotherapy (e.g., CBT, IPT), pharmacotherapy
Goedde et al. (2021) [43]	United States	“To examine depression outcomes in women receiving outpatient psychiatric services between 2007 and 2017 at a fully integrated OBMHC [Obstetric Mental Health Clinic] and to explore patient and obstetric team perceptions of OBMHC experiences.”	Chart/administrative data review	Pregnancy and postpartum	Depression	Collaborative care	Screening, assessment, psychiatric medication management, counselling, patient education, and follow-up
Green et al. (2016) [44]	United Kingdom	“To present the development of the Brockington Mother and Baby Unit.”	Program description	Pregnancy and postpartum	Psychosis, severe depression and anxiety	Inpatient Mother Baby Unit	Screening, assessment, specialist NP, care coordination between obstetrical and mental health clinicians, outreach contraceptive services, art therapy, CBT
Grote et al. (2015) [45]	United States	“To evaluate whether *MOMCare* is associated with improved quality of care and depressive outcomes compared to intensive public health maternity support services.”	RCT	Pregnancy	Major depressive disorder and dysthymia	Stepped care (*MOMCare)*	Screening, assessment, pretherapy engagement sessions, depression care management, brief IPT, psychiatrist consultations, pharmacotherapy, weekly multidisciplinary team meetings, case management
Harvey et al. (2018) [46]	Australia	“To describe the background, development, practice principles and evaluation findings of a nurse-led community model for perinatal mental health.”	Prospective cohort study	Pregnancy and postpartum	Depression and anxiety	Community PMH Clinic (Perinatal Wellbeing Service model)	Patient engagement, assessment, phone triaging, psychoeducation, psychotherapy (e.g., CBT), peer support, individualized treatment plans
Hauck et al. (2008) [47]	Australia	“To develop a framework for community mental health clinicians to improve the reproductive health outcomes for women with severe mental illness.”	Program description	Pregnancy	Serious mental illnesses (e.g., schizophrenia, bipolar disorder)	Community Mental Health Clinic (case management prevention)	Assessment, early detection and monitoring of pregnancy, small team approach, birth and postpartum preparation, patient education, individualized management plan, community referral process
Holmquist et al. (2021) [48]	United States	“To evaluate the integration of behavioral health services at a freestanding birth center.”	Survey	Pregnancy and postpartum	Depression	Collaborative care	Screening, support groups, counselling, psychotherapy (e.g., CBT, IPT) monthly multidisciplinary team meetings, embedded social worker, community referral process
Honikman et al. (2012) [49]	South Africa	“To describe a routine, programmatic, stepped-care model of maternal mental health care in a secondary level maternity hospital in Western Cape Province of South Africa Mowbray Maternity Hospital; linkage to University of Cape Town).”	Program description	Pregnancy and postpartum	Depression and anxiety	Stepped care (Perinatal Mental Health Project)	Screening, assessment, triaging, counselling, liaison with psychiatrist and allied health workers, ongoing follow-up, referral process, PMH staff training
Howard et al. (2006) [50]	United States	“To describe the development and implementation of a mother-baby day hospital service, and present preliminary data regarding treatment acceptability and effectiveness.”	Pre-post intervention	Pregnancy and postpartum	Depression	Day hospital	Referral process, intake assessment, triaging, individual and group psychotherapy (e.g., CBT, IPT), skill-building, pharmacotherapy, family psychoeducation, counselling, mother-infant dyad support, specialist consultations (e.g., lactation, infant development), onsite childcare, individualized treatment plans, aftercare psychotherapy group (post-discharge)
Howard et al. (2022) [51]	United Kingdom	To compare readmission rates of women admitted to MBUs and those of women admitted to other acute-care settings (generic psychiatric wards or crisis resolution teams).	Prospective cohort study involving interviews with patients and chart reviews	Postpartum	Depression and other mood disorders, psychosis, personality disorders, and eating disorders	Inpatient Mother Baby Unit	Parenting support and specialist parent–infant therapy, family/relationship therapy
Klatter et al. (2022) [52]	International (authors in Netherlands)	“To review antenatal mental health interventions and analyse the impact of collaborative care [… namely,] the effect of psychological or pharmacological interventions on the mental health of pregnant women with psychiatric symptoms (and psychosocial problems).”	Systematic review	Pregnancy	Depression, anxiety, insomnia, tocophobia	Collaborative care	A multi-professional approach to patient care, a structured management plan, scheduled patient follow-ups, enhanced interprofessional communication
Klawetter et al. (2021) [53]	United States	To describe an “integrated behavioral health program delivered in the Special Supplemental Nutrition Program for Women, Infants, and Children (WIC) and rooted in an infant and early childhood mental health (IECMH) framework”	Program description	Pregnancy and postpartum	Depression	Collaborative care (*Warm Connections*)	Screening, psychosocial support, brief behavioural interventions, relational interventions, referrals to community resources and mental health services
Kruper & Wichman (2017) [54]	United States	“To outline the development of an embedded multidisciplinary, PMH clinic comprised of psychiatry, psychology, and social work.”	Program description	Preconception, pregnancy, and postpartum	EPDS > 10, PMI symptoms, or identified need	Outpatient PMH Clinic	Screening, assessment, specialist consultation (social worker, psychiatrist, psychologist), pharmacotherapy, psychological interventions, patient education, staff training, individualized treatment plans
Kwitowski et al. (2024) [55]	United States	To describe the development and implementation of a proactive behavioural health team in inpatient obstetric care.	Program description	Pregnancy and postpartum	Mood and anxiety disorders, substance use	Collaborative care, proactive consultation-liaison psychiatric services	“(1) systematic screening for active mental health concerns, (2) proactive and preventive clinical interventions personalized to patient needs, (3) interdisciplinary team care, and (4) care integration with primary teams.”
LaRocco-Cockburn et al. (2013) [56]	Canada	“To compare, within two OB-GYN clinics, a collaborative care depression management model to usual care.”	RCT	Pregnancy and postpartum	Depression	Stepped care	Referral process, screening, assessment, psychotherapy (e.g., CBT, mindfulness), problem-solving therapy, pharmacotherapy, ongoing follow-up, multidisciplinary team discussions, psychiatrist consultations, community resources, patient education, PMH staff education
Lasater et al. (2021) [57]	Mali	“To identify a feasible and acceptable integrated care approach for the provision of maternal mental health care in rural Mali to help narrow the treatment gap and increase access to care.”	Qualitative – interviews and focus groups	Pregnancy and postpartum	Not explicit	N/A	PMH staff training, screening, referral process, group interventions, psychosocial care
Lever Taylor et al. (2021) [58]	United Kingdom	“To explore perinatal women’s experiences of specialist perinatal versus generic non-perinatal community mental health support.”	Qualitative—interviews with “women diagnosed with perinatal mental health difficulties”	Pregnancy and postpartum (although interviewees were all postpartum)	Depression, anxiety, personality disorder, psychotic disorders	Varied, including stepped care and collaborative care	For the perinatal mental health team group: tailored and specialist services; for all groups: home visits or flexible scheduling, coordinated services, referrals, 24/7 availability.
Lomonaco-Haycraft et al., (2019) [59]	United States	“To describe an integrated, sustainable infrastructure for the universal screening, assessment, and treatment of PMADs in alignment with national recommendations.”	Program description	Pregnancy and postpartum	Mood and anxiety disorders	Collaborative care (Integrated Behavioural Health model)	Screening, biopsychosocial assessment, behavioural health follow-up, psychological interventions (e.g., CBT, IPT, MBCT), psychoeducation, care coordination, referral process, transition support into primary care
Melville et al. (2014) [60]	United States	“To evaluate an evidence-based collaborative depression care intervention adapted to obstetrics and gynecology clinics compared with usual care.”	RCT	Pregnancy	Major depression and dysthymia	Collaborative care (Depression Attention for Women Now)	Patient engagement, screening, psychotherapy, pharmacotherapy, proactive outreach, ongoing follow-up and monitoring, weekly multidisciplinary team meetings, individualized treatment plans, staff training
Miller et al. (2020) [61]	United States	“To integrate lessons learned from perinatal collaborative care programs across the United States, recognizing the diversity of practice settings and patient populations, to provide guidance on successful implementation of a particular collaborative care program.”	Survey	Pregnancy and postpartum	Major depressive disorder, anxiety disorders, bipolar disorders, and substance use disorders	Stepped care	Screening tools, behavioural care managers, clinical care algorithms, referral process, patient engagement, individualized treatment plans, provider training
Miller (1996) [62]	United States	“To describe and review a program for prenatal and postpartum women with severe mental illness. To report the proportion of women who engage/do not engage with PMH services and their characteristics, as well as the strategies clinicians use to engage women.”	Program description	Pregnancy and postpartum	Severe and persistent mental illnesses (e.g., schizophrenia, major mood disorder)	Inpatient unit, outpatient clinic, and consult liaison	Screening, assessment, triage, referral protocols, individualized treatment plans, psychotherapy, pharmacotherapy, aftercare, sexual and reproductive planning, parenting assessment team, consult liaison team, staff training
Moore Simas et al. (2018) [63]	International	“To evaluate the feasibility, effectiveness, and acceptability of interventions that integrate depression care in outpatient obstetric practice.”	Systematic review	Pregnancy and postpartum	Depression	Varied	Screening, assessment, referrals, treatment titration, mental health consultation, co-location of maternity and behavioural health services, measurement-based treatment to target
Moses-Kolko et al. (2023) [64]	United States	“To examine the association of co-located behavioral health (BH) care with rates of OB-GYN clinician coding of BH diagnoses and BH medications.”	Chart/administrative data review	Pregnancy and postpartum	Depression and anxiety	Collaborative care	Screening, assessment, pharmacotherapy, interpersonal psychotherapy, and problem-solving treatment
Muzik et al. (2023) [65]	United States	“To describe the consultation and care arms of the Michigan Clinical Consultation and Care (MC3) program, a statewide program designed to facilitate access to perinatal mental healthcare for OB/Gyn patients.”	Program description	Pregnancy and postpartum	Depression, anxiety, substance use	Collaborative care (Michigan Clinical Consultation and Care [MC3] program)	Screening, assessment, psychoeducation, pharmacotherapy, psychotherapy, referrals to community resources
Myors et al. (2015) [66]	Australia	“To report the proportion of women who engage/do not engage with PMH services and their characteristics, as well as the strategies clinicians use to engage women.”	Mixed methods	Pregnancy and postpartum	Depression and psychosocial concerns	Not explicit – two specialist perinatal infant mental heath services	Building trust, therapeutic engagement, home visits, longer-term PMH services (2+ years)
Myors et al. (2013) [25]	International	“To identify professionals’ perceptions and experiences of collaboration and integration, when working with women, infants and families in the perinatal period who experience mental health problems, and to synthesize the findings across studies.”	Integrative review	Pregnancy and postpartum	Not explicit	Varied	Pathways between services, clinical guidelines, liaison workers for care continuity, co-location of maternity and mental health services, staff training
Oladeji et al. (2023) [67]	Nigeria	To report “on the impact of a cascade training (train-the-trainers) approach in improving the knowledge and attitudes of primary healthcare workers (PHCW) to perinatal depression”	Program description	Pregnancy and postpartum	Depression	Collaborative care: Task-sharing (WHO Mental Health Gap Action Programme-Intervention Guide [mhGAP-IG})	Staff training to screen and treat mental health issues in primary care settings
Oladeji et al. (2024) [68]	Nigeria	To report “the results of a task-shared approach for integrating care for perinatal depression (PND) within primary maternal and child healthcare (PMCH)”	Program description	Pregnancy and postpartum	Depression	Collaborative care: Task-sharing care collaborative care, WHO Mental Health Gap Action Programme-Intervention Guide (mhGAP-IG)	Staff training, screening, psychosocial interventions, low-dose medication
Petzold et al. (2021) [69]	Germany	To evaluate a “program that pools resources from psychiatric, obstetric, and pediatric departments as well as community and government agencies” based on treatment retention, psychosocial functioning, and abstinence.	Observational; program description	Pregnancy and postpartum	Methamphetamine use	Collaborative care	Family-centered care, shared decision-making, and patient participation in multidisciplinary meetings with specialists from psychiatric, obstetric, and pediatric departments as well as drug counseling and child welfare services.
Prom et al. (2022) [70]	International (authors in United States)	“To assess the effectiveness of interventions that integrate perinatal mental health care into routine maternal care to improve maternal mental health and infant health outcomes in LMICs [low- and middle-income countries].”	Systematic review	Pregnancy and postpartum	Depression and anxiety	Varied	Psychological, psychosocial, psychoeducational, adjuvant emotion/stress management integrated into primary care.
Puryear et al. (2019) [71]	United States	“To report on a quality improvement project designed to increase access to perinatal mental health services through universal screening for postpartum depression and facilitating referrals for evaluation and treatment, at a multi-site, integrated system of pediatric and obstetric practices.”	Program description	Pregnancy and postpartum	Depression	Collaborative care	Screening, mental health referral process
Sarkar et al. (2022) [72]	Uganda	“Qualitatively situating the extent to which integration of perinatal mental health care into maternal health care was considered desirable, possible and opportune within the existing policy and service-delivery environment in Uganda.”	Qualitative—interviews and focus groups	Pregnancy and postpartum	Depression	Collaborative care: task sharing	Sensitization and training for staff, including cross-learning across the health system (e.g., from HIV prevention clinics); screening; referrals
Selix et al. (2017) [73]	United States	“To present an interdisciplinary, cross-organizational approach coalescing diverse perspectives from those working across policy, research, training, primary care, and mental health in various disciplines to practice collaboratively to improve perinatal mental healthcare.”	Program description	Pregnancy and postpartum	Not explicit	Collaborative care (Maternal Mental Health NOW)	Screening, referral protocol, coordination between primary care and mental health services, technology-based tools, staff education, clinical policies
Soni et al. (2022) [74]	Australia	“To evaluate change in Health of the Nation Outcome Scale (HoNOS) scores from admission to discharge, readmission rates after 28-day and six months post-discharge, and factors associated with readmission in a Mother and Baby Unit (MBU).”	Cohort study based on chart/administrative data review	Postpartum	Depressive disorders, psychotic disorders, bipolar affective disorders, emotionally unstable personality disorder, bipolar affective disorders, anxiety disorders, eating disorders	Inpatient Mother-Baby Unit	24-hour care, treatment, referral
Tahchibana et al. (2019) [75]	Japan	“To examine the effects of a new multidisciplinary health service intervention program providing mental health care to mothers and children through pregnancy and childbirth.”	RCT	Pregnancy	Depression	Collaborative care	S creening, multidisciplinary care planning, individualized care plans, maternity/mental health/community consultations, defined roles and competencies, referral protocols
Tengelitsch et al. (2023) [76]	United States	To describe “the integration and role of masters-prepared behavioral health consultants (BHCs) within a state-wide psychiatry consultation program for children, adolescents, and perinatal women”	Program description	Pregnancy and postpartum	Mostly depression, some anxiety	Collaborative care, with some stepped care	Screening, assessment, triage, psychoeducation, referrals
Thomas et al. (2017) [77]	United States	“To describe a safety net system approach to address the psychosocial needs of at-risk women across the perinatal period.”	Program description	Pregnancy and postpartum	Major depression and trauma	Stepped care (Centering Pregnancy Model of Prenatal Care)	Co-located psychiatrist and maternity providers, infant-parent psychotherapy, social support, group prenatal care, community-based assistance with social needs, weekly interdisciplinary rounds, individualized labour plans, trauma and attachment-informed care
Truitt et al. (2013) [78]	United States	“To compare outcomes for women with depression receiving care through a collaborative model compared to routine primary care.”	Retrospective cohort study	Postpartum	Depression	Collaborative care (Collaborative Care Model Approach)	Patient registry, routine screening, depression symptom monitoring, weekly case reviews, mental health consultations, treatment guidelines, relapse prevention plans, patient education, care coordination
Wendt et al. (2021) [79]	International (lead author in United States)	“To synthesize the literature and develop guidance on supports needed for primary care and perinatal providers in screening, initial management, triage, and bridging treatment for perinatal bipolar disorder.”	Scoping review	Pregnancy and postpartum	Bipolar disorder	Varied	Screening, monitoring, treatment, referrals

ACT: acceptance and commitment therapy; BH: behavioural health; CBT: cognitive behavioural therapy; DBT: dialectical behavioural therapy; EMDR: eye movement desensitization and reprocessing; IPT: interpersonal therapy; MBCT: mindfulness-based cognitive therapy; NP: nurse practitioner; OBGYN: obstetrics and gynecology; RCT: randomized controlled trial.

PMH care models focused on specialized inpatient units, intensive hospital day programs, community clinics, and collaborative and stepped-care frameworks.

### Inpatient units

The inpatient care models included Mother Baby Units [44,51,74], a five-bed PMH unit [24], and a 6-bed PMH service embedded in a general adult psychiatry unit [62]. These in-hospital programs were designed to support birthing people requiring intensive care for severe PMIs (e.g., psychosis, severe depression) and featured interdisciplinary teams with specialized PMH training. While operational service aspects varied across programs, inpatient care models shared common features, including referral and intake pathways, integrated obstetrical and mental health assessment and treatment, and psychotherapy services. Of note, mother-baby units [44,50,74] and the PMH inpatient unit [24] facilitated mother-infant dyad admissions, while the general adult psychiatry unit [62] supported infant care in a nearby nursery. The average length of stay was reported as 22.35 days for one mother-baby unit [74] and 17 days for the general adult psychiatry unit [62].

### Intensive hospital day programs

Intensive PMH hospital day programs [41,50], positioned as alternatives to inpatient treatment, offered comprehensive, specialized, and interdisciplinary PMH care with the aim of minimizing disruptions to family and home life. In these models, care took place during daytime hours, with patients returning home at night and on weekends. Day treatment followed a flexible schedule and included a diverse range of individual and group treatment modalities, such as psychotherapy, pharmacotherapy, parental skill-building, and psychoeducation [41,50]. These programs also offered parent-infant therapy to promote responsive and attachment-focused interactions between parents and their infants [41,50].

### Community and outpatient clinics

Several articles described community and outpatient PMH care models [46,47,53,58,62,69]. These programs offered individualized and accessible consultation for and treatment of PMIs outside the hospital setting but differed in their purpose, team composition, and service offerings. As an example, the *Perinatal Wellbeing Service* led by nurses, provided non-urgent PMH assessment and brief intervention for individuals with perinatal mood and anxiety disorders [46]. In contrast, the *Case Management Prevention* framework utilized community mental health clinicians to support birthing people with serious mental illnesses (e.g., schizophrenia, bipolar disorder) through early detection of a problem and monitoring of pregnancy [47].

### Collaborative care frameworks

Among the most featured models, *collaborative care frameworks* assumed many forms but generally involved a multi-professional approach to establishing comprehensive and coordinated PMH care [37,40,43,45,52,55,57,60,64,65,69,71,72,76,78]. These models often consisted of systematic PMI screening at a maternity or primary care site, followed by connection to a PMH specialist when required. For example, in the *Partnership for Women’s Health* model [37], individuals were routinely screened using the Edinburgh Postnatal Depression Scale (EPDS), with scores above the cut-off triggering a referral to a PMH advisor who provided psychoeducation and referrals to appropriate resources. Collaborative PMH care models used diverse mental health interventions, including patient education [37,43,65,76], psychotherapy [45,57,65], pharmacotherapy, and psychiatrist follow-up [60,78].

In Nigeria and other low- and middle-income countries, *task sharing* was presented as a collaborative care approach for integrating mental health into primary, maternal, and child health care services [67,68,70]. This care delivery strategy aimed to improve access to evidence-based interventions in low-resource settings. Within this framework, non-specialist providers – individuals with no formal training in mental health, such as nurses, midwives, and community health officers – were trained to prevent, identify, and treat perinatal mental health concerns. Interventions included screening, psychoeducation, psychosocial interventions, and low-dose medication [67,68,70].

Other collaborative care models integrated mental health clinicians directly into existing maternity, community, or primary care clinics [24,38,39,43,55,56,59]. By co-locating mental health clinicians and perinatal care providers, these programs emphasized the use of “warm handoffs,” a seamless and timely linkage between perinatal patients and specialized mental health clinicians. That approach typically involved in-person introductions, real-time information sharing, and patient involvement in the clarification of treatment decisions. These frameworks also incorporated a structured mechanism to promote scheduled communication between maternity and mental health care providers to facilitate ongoing PMH monitoring and care planning discussions.

### Stepped-care frameworks

Finally, eight articles (17%) described stepped-care models, an approach focused on matching the intensity of intervention to individual care needs [28,42,45,49,56,58,61,76]. These models typically offered low-intensity interventions to start mental health care and provided progressively more intensive interventions as needed. As illustrated in Gjerdingen et al.’s [42] “Stepped Care Treatment of Postpartum Depression,” most frameworks used screening and assessment to inform the recommendation of an initial treatment step. In general, low-intensity treatments consisted of psychosocial and community interventions (e.g., peer support, self-help), moderate-intensity treatments consisted of psychological (e.g., psychotherapy, counselling) and pharmacotherapeutic interventions (e.g., antidepressant medication), and high-intensity treatments consisted of specialized psychiatric interventions such as PMH day treatment and inpatient hospitalization. All stepped-care models emphasized the need for continuous monitoring to assess treatment effectiveness and inform care modifications.

### Key elements of integrated models of PMH care

**Fig 3:**
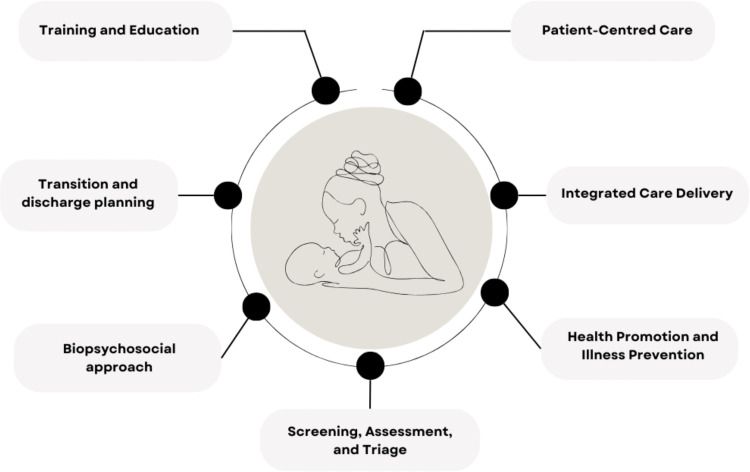
Key elements of integrated PMH care.

Fig 3 provides a visual representation of the key elements of integrated models of PMH care identified in our scoping review. All 47 articles featured PMI *screening* and *integrated approaches to care delivery*, while most articles also described the importance of *patient-centred care, PMH-trained clinicians,* and a *biopsychosocial approach to treatment*. The concept of *health promotion and illness prevention* largely emerged in discussions related to preconception planning and pre-existing mental illness, while *transition and discharge planning* was emphasized in the context of stepped-care frameworks*.* Details related to each care element are provided below.

### Screening, assessment, and triage

Screening, a key element of all care models, was often described as a critical measure in the prevention, detection, and management of PMIs [54] with the potential to improve health and social outcomes for perinatal people and their families [63]. Additionally, many programs noted that routine perinatal and well-child visits offered convenient opportunities to screen pregnant and postpartum people in familiar care settings [61,62]. With respect to screening tools, the EPDS was the most cited (n = 24); however, the Patient Health Questionnaire-9 (PHQ-9) [42,45,56,60,61,63,65,70,73,76,78,79] and Beck Depression Inventory (BDI) [63,70] were also commonly referenced. There was no consensus on the optimal time in which to conduct screening, though many articles reported screening during the third trimester of pregnancy (e.g., Lomonaco-Haycraft et al. [59]) and/or within the first three months postpartum (e.g., Baker-Ericzen et al. [37]; Kruper & Wichman [54]).

Most care models articulated a protocol with which to respond to screening results. This typically involved a care provider reviewing results with the patient, offering in-the-moment resources (e.g., educational material, resource lists, etc.), and referring to a PMH specialist (e.g., mental health advisor, perinatal psychiatrist, etc.) when appropriate (e.g., Bowen et al. [39]; Lomonaco-Haycraft et al. [59]). Some programs, as seen in the *Partnership for Women’s Health* [37] and the *University of Illinois at Chicago’s Women’s Program* [62], also detailed procedures related to subsequent assessment and triaging. These processes aimed to further characterize PMI (e.g., nature and severity of symptoms), identify potential safety risks, determine goals of care, and inform the type and timing of follow-up services.

### Integrated care delivery

All articles featured integrated care delivery systems, encompassing structures, teams, and processes designed to promote clinical collaboration, coordination, and continuity across the care continuum. While programs varied in their overall approach to care integration, most involved some version of shared information systems [78], multidisciplinary teams [75], standardized referral and intake pathways [50], ongoing symptom and outcome monitoring [42], and PMH decision support tools [47,61]. Care coordination – the act of organizing patient care activities across multiple disciplines and specialists – also took many forms, including warm hand-offs [59,61], case management [60], structured team meetings [56,77], and collaborative care planning [24].

### Patient-centred care

Many PMH care models endorsed a patient-centred approach respecting an individual’s unique preferences, values, and circumstances throughout the care process (e.g., Harvey et al. [46]; Miller et al. [61]; Petzold et al. [69]). Care teams were particularly invested in sharing information with patients in a timely manner to support personalized care decisions. As an example, the *Mother Baby Connections* program used a Patient Navigator to communicate with patients between intensive day treatments to address concerns and facilitate referrals as required [41]. Other programs actively engaged patients in identifying care goals and developing personalized care plans [24,60,61,75].

### Training and education

PMH staff training was consistently identified as essential to integrated care. Numerous articles suggested that clinicians, across disciplines, should receive opportunities to develop the specialized skills, knowledge, and confidence to effectively manage the unique challenges and complexities of PMIs [24,25,41,49,56,57,60–62,66–68,71–73]. While some care models suggested training related to specific tasks such as administering PMI screening [71], others advocated for training inclusive of clinical (e.g., assessment and management) and non-clinical (e.g., practical aspects of collaborative work) components of integrated care [25]. Approaches to training were wide-ranging and included traditional instruction [41,42,60] and clinical supervision [41,61,62]. In the context of task-shared care [67,68], cascade training offered an efficient model for upskilling care providers in the identification and management of PMH concerns. This approach leveraged “master trainers” to train smaller groups of non-specialists to deliver evidence-based mental health interventions in low-resource settings.

### Biopsychosocial approach to treatment

Although the PMH care models included in the review offered many types of clinical interventions, nearly all articles described the importance of a biopsychosocial approach to treatment to address the complex physical, psychological, and social aspects of PMI – recognized as interconnected factors that impact individual experiences of health and illness. Frequently, treatment plans specified various intervention options adaptable to a patient’s needs, strengths, and preferences [61]. These interventions generally included psychoeducation, psychotherapy (e.g., cognitive behavioural therapy [CBT], interpersonal therapy [IPT], parent-infant therapy), and pharmacotherapy [41,46]. Additionally, most models emphasized the need for ongoing assessment and monitoring to evaluate treatment effectiveness and inform treatment modifications [49,56,78].

### Health promotion and illness prevention

In the context of preconception care and care of individuals with pre-existing mental illnesses, several articles discussed health promotion and illness prevention. Health promotion was broadly described as proactive strategies to enhance and maintain an individual’s overall well-being and ability to cope with adversity, while illness prevention was framed as strategies to address risk factors related to PMI recurrence or exacerbation [47,77]. In both domains, PMH education was leveraged as an important strategy to discuss PMI prevalence, risk factors, and symptoms; potential impacts of untreated or undertreated illness; strategies to promote mental health; and access to community health and social resources [76]. Care models emphasized the importance of pregnancy monitoring alongside individualized care planning for individuals with pre-existing mental illness, including prenatal care, labour management, detection of illness, and optimization of protective factors [47].

### Transition and discharge planning

Finally, several articles discussed transition and discharge planning – the coordination, communication, and mobilization of resources required to support patients moving between levels of care or clinical settings. This type of planning was most frequently reported in the context of stepped-care frameworks and often encompassed written or verbal communication about care plans between clinical teams [61]. The care plans addressed patient goals, interventions and treatment, safety planning, and social needs [50].

## Discussion

This scoping review advances PMH service literature by summarizing peer-reviewed evidence about integrated approaches to PMH care. We identified 45 articles encompassing a wide range of PMH care models, including specialized inpatient units, intensive hospital day programs, community clinics, and collaborative and stepped-care frameworks. Analysis of these models further revealed seven key elements of integrated care: (1) screening, assessment, and triage; (2) integrated care delivery; (3) patient-centred care; (4) a biopsychosocial approach to treatment; (5) PMH-trained clinicians; (6) health promotion and illness prevention; and (7) transition and discharge planning.

Our findings indicate a growing interest, over the past decade, in the development of integrated PMH programs in North America and Australia. Many of these programs involved cross-specialty (e.g., obstetrics, mental health, primary care) collaboration through the coordination of a range of health services historically operating independently of each other. By linking clinical teams and care processes through various modes of integration (e.g., shared information systems, referral pathways, case management), the evidence suggests that integrated PMH care models can improve treatment accessibility, care continuity, and patient experience for perinatal people and their families [25,60,63].

With respect to terminology, we found no single definition of integrated care. The term referred to multiple interventions, strategies, and frameworks applied across a variety of clinical settings. Integrated care was also frequently conflated with concepts such as *collaborative care* [78]*, shared care* [17], and *multi-disciplinary care* [77], and pursued to achieve a diverse range of outcomes, including enhanced maternal functioning [41], improved symptom severity [24], higher patient satisfaction [48] and increased rates of mental health treatment [42]. Recognizing that terminology plays a critical role in shaping the design and delivery of health care services [80], it might be useful to establish an operational definition of integrated care that fosters shared understanding, meaningful application, and comprehensive evaluation in future endeavours [81].

Our results are consistent with other evidence syntheses of integrated care. Firstly, as multiple reviews have established [1,82,83], we did not identify a single “best” approach to integrating care. PMH care models often use multiple interventions to improve care continuity and coordination [25,63]. It was also difficult to consider the impact of any individual care element given that most PMH models were implemented as integrated, whole programs. Secondly, patient-centred care emerged as a core feature of integration. In line with many multi-morbidity models [4,83–86], PMH programs prioritized engaging patients in care decision-making to promote the personalization of integration strategies, care goals, and treatment plans [46,61]. Thirdly, key elements of integration appeared to operate at multiple levels of service provision. That is, care components targeted micro (individual/clinical), meso (organizational/service), or macro (system) levels of integration, with the majority of elements focused on micro-level approaches (e.g., screening, case management, shared information systems, etc.). The emphasis on clinical integration reflects well-documented complexities related to system-level redesign, such as the development of new social services and the implementation of health policies [1,83,84].

Finally, it is worth noting that the care models included in our review predominantly focused on identifying and managing perinatal depression. The prioritization of depression likely reflects its high prevalence following childbirth [87,88]; however, there is increasing recognition that a more expansive view of PMH care is required [89,90]. Perinatal people with severe mental illnesses, such as schizophrenia and bipolar disorder, are at significantly higher risk for obstetrical and neonatal complications [91–94]. Many individuals, with these diagnoses, delay antenatal care and require additional support to manage psychosocial concerns [89]. In light of available evidence, our scoping review suggests that these illnesses are both under-researched and underserved by existing PMH programs. As such, there may be substantial value in the development and evaluation of integrated care models that support the full spectrum of PMIs, including care of individuals with chronic severe mental illness and those with new-onset psychosis [89,94].

This scoping review offers an important overview of integrated PMH care, mapping the breadth and depth of an evolving body of literature to determine gaps in evidence and identify areas for future empirical work. However, there are several limitations to acknowledge. Our review was restricted to peer-reviewed articles published in English and did not include grey literature. In addition, our broad search strategy did not target individual elements of integrated care; therefore, relevant research related to specific integration strategies may have been missed. Lastly, given the complex and overlapping nature of integrated care interventions, it was not possible to appraise the importance of any individual element despite the frequency with which it may have appeared in the literature.

## Conclusion

To our knowledge, this review is the first to examine care models and key elements of integrated PMH care. Our findings suggest that while integration can take many forms and apply to different levels of service provision, integrated PMH care – at its core – aims to enhance care quality and continuity through patient engagement, trained staff, health promotion and illness prevention, coordinated care processes, and a biopsychosocial approach to treatment. Given the current emphasis on perinatal depression care and the considerable morbidity associated with severe mental illness, our results also indicate a need for PMH care models that support the full spectrum of PMIs. We further advocate for a greater focus on macro-level approaches to integration to facilitate the implementation of integrated PMH care at scale. Finally, we see value in establishing Canadian consensus on the key elements of integrated PMH care identified in this review, including the perspectives of clinicians and people with lived PMI experience.

## Supporting information

S1 Table Search strategy example (EMBASE). (DOCX)

S1 Checklist PRISMA-ScR checklist. (PDF)
