# Supplementary material for: Models and key elements of integrated perinatal mental health care: A scoping review
Source: PLOS Ment Health. 2025 Mar 17;2(3):e0000164. doi: 10.1371/journal.pmen.0000164 (PMC12798406; doi:10.1371/journal.pmen.0000164)
Supplement: S1 Table — (DOCX) [file pmen.0000164.s001.docx]

S1 Table: Search Strategy Example (EMBASE)

| Search ID# | Search Terms | Results |
| --- | --- | --- |
| 1 | integrated health care system/ | 11,797 |
| 2 | ("integrated care" or "continuity of care" or "shared care" or "coordinated care" or "multidsciplinary care" or "interdisciplinary care" or "care continuation").mp. [mp=title, abstract, heading word, drug trade name, original title, device manufacturer, drug manufacturer, device trade name, keyword, floating subheading word, candidate term word] | 22,845 |
| 3 | perinatal period/ | 35,861 |
| 4 | pregnant woman/ or pregnancy/ | 660,811 |
| 5 | birth/ | 18,389 |
| 6 | obstetric delivery/ | 12,900 |
| 7 | ("prenatal" or "antenatal" or "antepartum" or "pregnan*" or "perinatal" or "peripartum" or "puerperal" or "intrapartum" or "birth" or "childbirth" or "postnatal" or "postpartum").mp. [mp=title, abstract, heading word, drug trade name, original title, device manufacturer, drug manufacturer, device trade name, keyword, floating subheading word, candidate term word] | 1,487,869 |
| 8 | *mental health/ | 42,298 |
| 9 | mental disease/di [Diagnosis] | 20,201 |
| 10 | mental health care/ | 29,455 |
| 11 | depression/ or postnatal depression/ or perinatal depression/ or antenatal depression/ | 387,116 |
| 12 | mood disorder/di [Diagnosis] | 3,080 |
| 13 | bipolar disorder/di [Diagnosis] | 5,322 |
| 14 | schizophrenia/di [Diagnosis] | 14,679 |
| 15 | acute psychosis/ or psychosis/ or affective psychosis/ or schizoaffective psychosis/ | 101,025 |
| 16 | ("mental health" or "mental disorder*" or "psychiatr*" or "depress*" or "anxiety" or "schizophrenia" or "psychosis").mp. [mp=title, abstract, heading word, drug trade name, original title, device manufacturer, drug manufacturer, device trade name, keyword, floating subheading word, candidate term word] | 1,620,635 |
| 17 | 1 or 2 | 33,093 |
| 18 | 3 or 4 or 5 or 6 or 7 | 1,488,643 |
| 19 | 8 or 9 or 10 or 11 or 12 or 13 or 14 or 15 or 16 | 1,625,255 |
| 20 | 17 and 18 and 19 | 285 |
